# A custom tailored, evidence-based, theory-informed intervention for healthcare professionals to prevent burnout (LAGOM): study protocol for a pragmatic randomized controlled trial

**DOI:** 10.1186/s13063-024-08491-1

**Published:** 2024-09-27

**Authors:** Anna K. Koch, Marleen Schröter, Julia Berschick, Julia K. Schiele, Martin Bogdanski, Melanie Steinmetz, Wiebke Stritter, Andreas Voss, Georg Seifert, Christian S. Kessler

**Affiliations:** 1 Charité Competence Center for Traditional and Integrative Medicine (CCCTIM), Charité – Universitätsmedizin Berlin, Corporate Member of Freie Universität Berlin, Humboldt-Universität Zu Berlin and Berlin Institute of Health, Berlin, Germany; 2 Department of Prevention, Integrative Medicine and Health Promotion, Charité – Universitätsmedizin Berlin, corporate member of Freie Universität Berlin, Humboldt-Universität Zu Berlin, and Berlin Institute of Health, Berlin, Germany; 3 Department of Internal Medicine and Nature-Based Therapies, Immanuel Hospital Berlin, Berlin, 14109 Germany; 4 Institute of Biomedical Engineering and Informatics (BMTI), Technische Universität Ilmenau, Ilmenau, Germany; 5 Departamento de Pediatria, Universidade de São Paulo, Faculdade de MedicinaInstituto de Tratamento Do Câncer Infatil (ITACI), São Paulo, Brazil; 6 Institute of Social Medicine, Epidemiology and Health Economics, Charité – Universitätsmedizin Berlin, corporate member of Freie Universität Berlin, Humboldt-Universität Zu Berlin, and Berlin Institute of Health, Berlin, Germany

**Keywords:** Burnout, Prevention, Healthcare professionals, Study protocol, Effectiveness, Mind–body medicine

## Abstract

**Background:**

Healthcare professionals in hospitals are exposed to a high level of professional stress, time pressure, workload, and often times poor organizational support. This makes them especially susceptible to burnout. In this pragmatic randomized controlled trial, we test the hypothesis that participation in a 9-week program (*LAGOM*) that was designed in close collaboration with healthcare professionals, incorporating both individual and organizational aspects reduces emotional exhaustion in healthcare professionals (primary outcome).

**Methods:**

Eighty four nurses and physicians working at the Charité – Universitätsmedizin Berlin and the Immanuel Hospital Berlin are automatically randomized to the *LAGOM* program (*n* = 42) or to usual care (*n* = 42) in a one-to-one allocation rate. The primary outcome emotional exhaustion is measured by the Maslach Burnout Inventory—Human Services Survey at baseline, post-intervention, and 1-month follow-up via an online survey. Secondary outcomes include depersonalization, personal accomplishment, subjective stress, mental well-being, self-care, self-efficacy, working conditions, mindfulness, and adverse events. Electrophysiological measures for heart rate variation analysis are captured. The PRECIS-2 tool is used to characterize the degree of pragmatism in our trial. Data analysis and primary intention-to-treat analysis using repeated measures analysis of variance are performed blind to intervention allocation. Per-protocol, subgroup, and secondary outcome analyses are conducted exploratively. An advisory board consisting of various stakeholders accompanies the study process.

**Discussion:**

If *LAGOM* proves to be effective in reducing symptoms of burnout, the program could make an important contribution to tackling the problem of the very high burnout rates among healthcare professionals and become an integral part of preventive services offered by hospitals.

**Trial registration:**

German Clinical Trials Register, DRKS00034060. Registered 31 May 2024.

**Supplementary Information:**

The online version contains supplementary material available at 10.1186/s13063-024-08491-1.

## Background

Burnout among healthcare professionals, particularly those employed in hospital settings, is a pervasive and concerning issue that demands systematic investigation and targeted interventions. The rising prevalence of burnout has far-reaching consequences, affecting not only the well-being of healthcare professionals but also patient safety, the quality of care, and the overall functionality of healthcare systems [1–9]. In the workplaces of healthcare professionals, stressors such as a high workload, dealing with suffering patients, and other workplace-related conflicts are among the greatest risk factors for developing burnout [10–12]. The COVID-19 pandemic has further intensified this [13, 14]. The 2024 Medscape National Physician Burnout and Depression Report estimated a burnout rate of approximately 49%, suggesting much higher values for burnout than in the general population [15]. Europe and especially Germany are no exception to these high levels [16–18]. Understanding the multifaceted nature of burnout necessitates a comprehensive and tailored approach to interventions.

There are different person-directed interventions and organization-directed interventions that can result in small but meaningful reductions in burnout scores [19, 20]. However, the field has made limited progress in addressing burnout. Conclusions suggest that individual and organizational solutions should ideally be combined to achieve greater improvements in well-being, that interventions should be easily accessible in the workplace, and that employees should participate in the design and implementation process to address unique organizational and staff needs [20–23].

We aim to refine and improve the design of burnout prevention interventions for healthcare professionals working in a hospital setting. To this end, incorporating findings from previous interventions and actively involving various stakeholders, including future participants of the intervention, the *LAGOM* program was developed. *LAGOM* integrates individual prevention through mind–body medicine (MBM) with prevention aspects at the organizational level. It takes place within working hours and its implementation and evaluation is fully supported by the management of the two participating hospitals. Following the feasibility testing of *LAGOM* (study registration: DRKS00032014), a high-quality randomized controlled trial (RCT) is now required to test the effectiveness of the program, to evaluate the sustainability of the program during the longer follow-up periods and to obtain a more comprehensive overview of the potential impact of the program on the working environment of healthcare professionals.

## Objectives

This study aims to evaluate whether a custom-tailored, evidence-based, theory-informed intervention for healthcare professionals working at hospitals (i.e., the *LAGOM* program) reduces burnout symptoms and stress. The primary hypothesis is that compared to usual care, *LAGOM* leads to a reduction in emotional exhaustion, one of the main burnout symptoms, after the intervention.

## Methods

### Design

The study is a pragmatic RCT with two parallel arms, a one-to-one allocation ratio and two study sites. Ethics approval was granted from the Ethics Committee of the Charité – Universitätsmedizin Berlin on May 29th 2024 (EA4/061/24). The trial has been registered with German Clinical Trials Register on May 31st 2024 (https://drks.de/search/de/trial/DRKS00034060). We use the SPIRIT guidelines to guide reporting of the trial protocol [24]. The study is performed at two study sites: the Charité – Universitätsmedizin Berlin, Berlin, Germany, and the Immanuel Hospital, Berlin, Germany. The study organization takes place at the Charité – Universitätsmedizin Berlin. In the event of necessary protocol adaptations, all parties concerned will be informed promptly.

### Eligibility criteria

As the present study is a pragmatic trial, inclusion and exclusion criteria are limited as far as possible. Inclusion criteria are:

- Working healthcare professionals, actively practicing medicine or nursing at Charité – Universitätsmedizin Berlin or Immanuel Hospital, Germany
- 18 years or older
- Completed written informed consent
- Proficient in German language

Exclusion criteria are:

- Currently clinically diagnosed burnout syndrome according to ICD-11 (QD85 “Burnout”)
- Purely administrative position
- Pregnancy

The research nurse will confirm eligibility regarding all aspects during the screening process.

### Intervention

The *LAGOM* program (*L*ongterm *A*pproach and *G*uidelines for *O*ccupational Mental Health with *M*BM) is developed based on the intervention mapping approach (IMA) by Eldredge et al. [25]. In addition to the core project team, which consists of physicians, psychologists, nutritionists, sports scientists, and biosignal experts, various advisory boards are involved in the development of the intervention. A group of stakeholders consisting of physicians and nurses, the intervention’s intended audience, is also part of the advisory boards. The advisory boards provided ongoing feedback during the development phase of the intervention, which was incorporated into the program and tailored specifically to the needs of the target group. The *LAGOM* program has already been subjected to a feasibility test on 24 test participants, revised on this basis and adapted even better to the needs of the nursing staff and physicians. The *LAGOM* program and the IMA steps will be described in detail in a later publication. The content and structure of the *LAGOM* program are briefly outlined below.

**Fig. 1:**
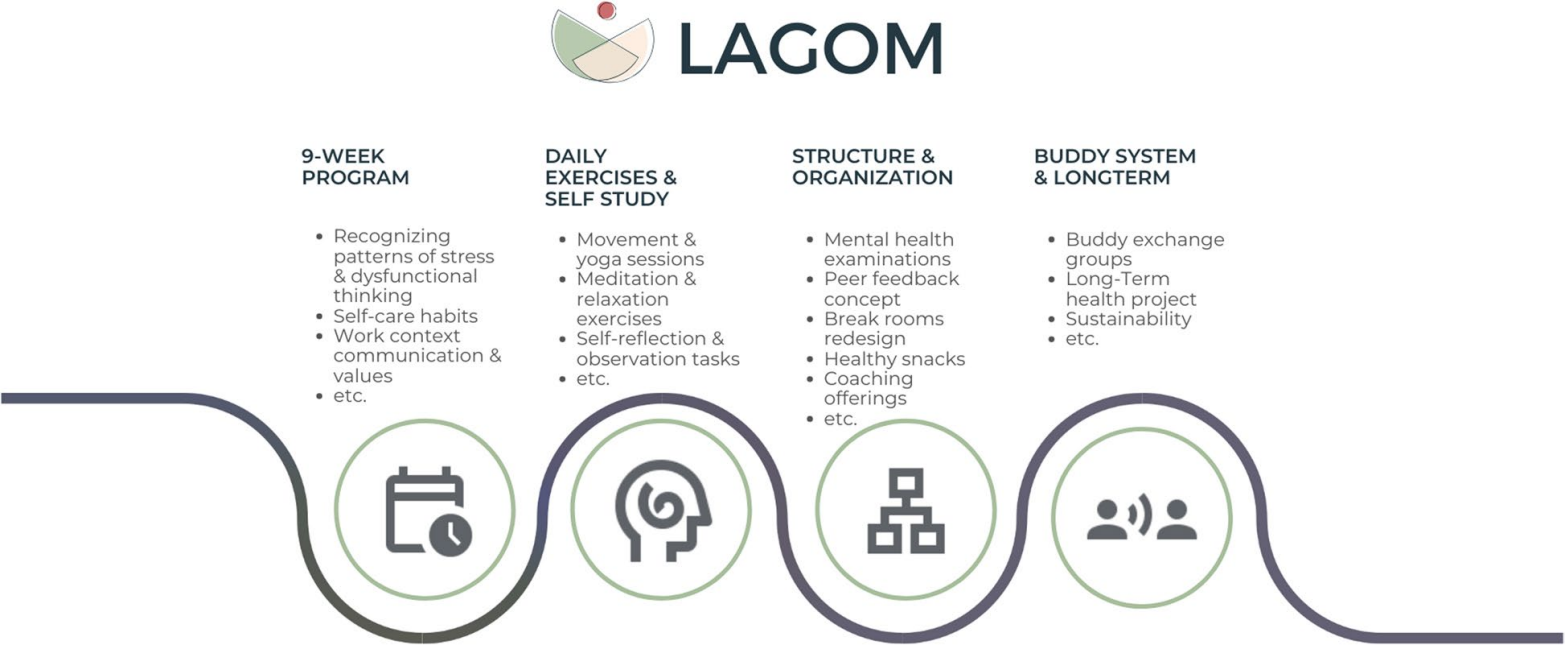
LAGOM overview

The *LAGOM* program is conducted over a period of 9 weeks with one session per week lasting between 3 h (first and last session) and 90 min (7 sessions, Fig. 1) with an ideal group size of 10 to 15 participants. The course units alternate between online and face-to-face sessions. The online sessions are held via Microsoft Teams. The face-to-face meetings take place in a room rented specifically for this purpose at the locations of the two study centers. The behavioral prevention part of the 9-week program is largely based on elements of MBM. MBM emphasizes the connections between thoughts, feelings, behaviors, and the body and strives to promote health and well-being and to treat disease by addressing mental, emotional, social, and psychological factors. These skills promote self-awareness, self-care and resilience—factors that are crucial for emotional well-being and may also reduce burnout symptoms [26, 27]. The weekly *LAGOM* sessions are led by three experienced MBM therapists, two physicians and one systemic coach. One trainer is leading one cohort respectively. The MBM therapists receive training in the LAGOM program by one of the core members of the project team (JS) before study start and a detailed intervention protocol of the course content with a set schedule for topics and exercises to ensure generalizability across cohorts. In addition, during each cohort, one supervision meeting with all trainers and core members of the project team (JB and MS) is provided. Trainers are instructed to follow the protocol and to document any changes made to it. Sessions follow a consistent structure, containing a psycho-educational part on various topics, practical exercises and interactive exchange, a relaxation exercise, and conclusion of the session. The sessions address topics such as recognizing stress patterns, dysfunctional patterns of thinking, establishing self-care habits, goal setting, behavior change techniques, communication and values in the work context, complemented by practical exercises such as mindfulness exercises, short movement/yoga sessions, meditation and relaxation exercises, self-reflection, and observation tasks. Participants are motivated to practice the newly learned skills for 5–15 min at home or during their daily work. As maintenance and follow-up, there are buddy exchange groups for weekly dialogs and an individual health goal (project) beyond the course period. This shall lead to a sustainable individual routine, which the participants then maintain independently. In addition, structural and organizational aspects are addressed and offered on a weekly basis in dialog with the hospital’s individual wards. Participants receive weekly e-mails with impulses on structural level, such as mental health examinations in staff appraisals, the peer feedback concept, the redesign of break rooms, healthy snacks, coaching offers, an interdisciplinary lunch break (“happy hour”), as well as structural health offers to improve recovery and well-being. As the needs and requirements vary between the individual wards, study participants together with their ward team can decide which impulses are useful for them and which they want to pursue.

### Control

The control group receives usual care. Usual care is the comparator of choice in pragmatic trials [28]. It consists of the already existing courses for burnout prevention and stress reduction at the Charité – Universitätsmedizin Berlin or the Immanuel Hospital Berlin. This includes 1-day classroom courses on stress regulation or building resilience, as well as various online offers, for example on the topic of mindfulness, from various (external) providers. As participation in such courses is not mandatory, participants who do not participate in such courses are also included as controls. After the study period, participants of the control group are offered to participate in the *LAGOM* program as well. No concomitant intervention is permitted or prohibited during the trial in both groups.

### Outcomes and data collection

Data is collected before (baseline, week 0), post-intervention (week 10), and follow-up 1 (week 14). Attrition, intervention adherence and adverse events are collected on a weekly basis (weeks 1–9) by the trainers of the MBM course via attendance lists. Table 1 shows an overview of the evaluation plan. Based on the IMA [25], the structure of the effectiveness analysis is oriented toward the four categories (1) quality of life, (2) behavior, (3) environmental conditions, and (4) determinants.

**Table 1:** Evaluation plan for the *LAGOM* program

Study period							
	**Measures**	**Sources**	**Pre-intervention**	**Baseline**	***LAGOM*- program**	**Post-intervention**	**Follow-up**
				**Week 0**	**Weeks 1–9**	**Week 10**	**Week 14**
**Enrollment**							
Eligibility Screen	Eligibility checklist	Participants	X				
Informed Consent & Assent		Participants	X				
**Data collection**							
Sociodemographics	Survey	Participants		X			
Attrition	Study records	Research staff			X		
Intervention adherence	Study records	Trainers			X		
Adverse events	Study records	Trainers Participants			X	X	
**Effectiveness evaluation**^**b**^							
** Quality of life**							
Burnout symptoms	Questionnaire (MBI-HSS)	Participants		X		X	X
Well-being	Questionnaire (WHO-5)	Participants		X		X	X
Subjective stress	Questionnaire (PSS-10)	Participants		X		X	X
** Behavior**							
Self-care	Questionnaire	Participants		X		X	X
** Environmental conditions**							
Working conditions	Questionnaire (ISAK-K)	Participants		X		X	X
** Determinants**							
Self-efficacy	Questionnaire (BSW-5-REV)	Participants		X		X	X
Mindfulness	Questionnaire (FMI 13-r)	Participants		X		X	X
**Electrophysiological measures**							
Variability analysis (HRV, PWV, RESP, EDA) of ANS functions represented in stress indicators from cardio-vascular variability and sympatho-vagal balance	Biosignal recordings including ECG, PPG, RESP, and EDA	Participants		X		X	

*MBI-HSS* Maslach Burnout Inventory Human Services Survey, *FMI* Freiburg Mindfulness Inventory, *WHO-5* WHO-5 – Well-Being Index, *PSS-10* Perceived Stress Scale, *ISAK-K* Instrument for Stress-Related Job Analysis for Hospital Physicians short version, *BSW-5-Rev* Scale for measuring occupational self-efficacy expectation [Skala zur Messung der beruflichen Selbstwirksamkeitserwartung], *HRV* heart rate variability, = pulse wave variability, *RESP* respiration activity, *EDA* electrodermal activity, *ANS* autonomic nervous system

^b^Like the development of the *LAGOM* program, the effectiveness evaluation will be based on Eldredge´s intervention mapping approach (IMA); accordingly, a consideration of the four main components in IMA (quality of life, behavior, environmental conditions and determinants) is planned

#### Quality of life

The primary outcome is *Burnout – Subscale Emotional exhaustion* as measured by the Maslach Burnout Inventory Human Services Survey (MBI-HSS) [29], German version. The decision to use emotional exhaustion as the primary outcome was made in several meetings of the core team. The MBI-HSS addresses three burnout subscales (emotional exhaustion, depersonalization and personal accomplishment) with 22 items that can be rated on a scale ranging from 0 = *never* to 6 = *every day* with higher scores indicating a higher level of burnout except for personal accomplishment where higher scores indicate less burnout. Emotional exhaustion is captured with nine items, resulting in scores between 0 and 54. *Depersonalization* is captured with five items, resulting in scores between 0 and 30. *Personal accomplishment* is captured with eight items, resulting in scores between 0 and 48. Depersonalization, personal accomplishment, and the c*omposite score* are secondary outcomes. Validity and reliability of the MBI have been demonstrated to be good or acceptable (Cronbach’s *α* > 0.7 for all subscales) [29–31]. *Well-being* is assessed using the WHO-5 – Well-Being Index [32]. This five items questionnaire assesses emotional well-being over the last 2 weeks on a 6-point scale ranging from 0 = *at no time* to 5 = *all of the time* with higher scores indicating higher well-being. Validity and reliability of the WHO-5 have been demonstrated to be good with reliability ranging between 0.83 and 0.93 [33]. *Subjective stress* is assessed using the Perceived Stress Scale (PSS-10; [34]), German version [35]. The PSS-10 measures the degree to which life in the past month has been experienced as unpredictable, uncontrollable, and overwhelming on a 5-point scale ranging from 0 = *never* to 4 = *very often* with higher scores indicating higher stress.

#### Behavior

Self-care is measured using 16 items suitable for the *LAGOM* program, which are taken from a non-validated questionnaire, the “Self-Care Questionnaire” and will be translated into German (supplement).

#### Environmental conditions

The ISAK (Instrument for Stress-Related Job Analysis for Hospital Physicians) identifies problem areas in physicians’ working conditions. The short version (ISAK-K), consisting of 14 scales and 30 items, captures the stressors and resources of clinicians [36]. It has internal consistencies of Cronbach’s alpha = 0.70–0.95 [36]. It is also administered to nurses and the wording adjusted accordingly, e.g., for the item “In our department, inexperienced medical colleagues have ample opportunity to benefit from the knowledge and skills of experienced colleagues” the term “nursing colleagues” is added.

#### Determinants

*Occupational self-efficacy* is assessed by the BSW-5-Rev (German: Skala zur Messung der beruflichen Selbstwirksamkeitserwartung; scale for measuring work-related self-efficacy) [37]. The scale consists of five items that can be rated on a scale ranging from 1 = *completely disagree* to 4 = *completely agree* with higher scores indicating higher self-efficacy. Research has shown good construct and criterion validity and acceptable internal consistency (Cronbach’s *α* = 0.73 for employee version) [37]. *Mindfulness* is assessed using the Freiburg Mindfulness Inventory [38, 39] German revised version 13-r [40]. The scale consists of 13 items that can be rated on a scale ranging from 1 = *rarely* to 4 = *almost always* with higher scores indicating more mindfulness. The German version is a psychometrically sound and valid instrument with McDonald’s Omega = 0.88 [40].

#### Adverse events

Details of adverse events or harms are documented by participants and/or trainers when reported to them on an a priori designed structured form and will be monitored by the research team.

The Project Management Team will meet weekly to review trial conduct. In case any changes need to be made to the initial ethics application or in case of adverse events, the Ethics Committee of the Charité – Universitätsmedizin Berlin will be informed to review conduct.

Long-term follow-ups are also planned to be collected 6 and 12 months after the intervention via the online survey sent to each participant, but due to the limited project duration and funding constraints these are not part of this manuscript and will be captured and published at a later date, subject to further funding of the project.

The following baseline data is collected prior to randomization to describe the sample and conduct exploratory subgroup analyses: (1) participants’ prior experience with MBM, (2) expectations with regard to effectiveness, (3) work location (Charité or Immanuel), (4) other baseline data: age, gender, occupation, full-time, additional function within the scope of employment, cultural background (optional), shift work, private care-responsibilities, work experience, leadership responsibility, and existing or past diagnosis of a mental illness.

*Intervention adherence* is defined as the number of sessions attended and will be documented by the MBM trainers via attendance lists. *Attrition* is defined as participant dropout over time, recorded by the study team in the study records. Reasons for dropout are documented as well.

### Electrophysiological measures

Non-invasive electrophysiological measurements are recorded at baseline and after the end of the intervention program at each study site. Analyzing biosignals and their resulting indices allow assessments to be made about the autonomic regulation and more specifically the sympatho-vagal balance. For example, cardio-vascular estimates of the autonomic nervous system (ANS) functions are represented by heart rate variability (HRV) and pulse wave variability (PWV) measures, which have been established and further developed since many years [41–44]. Analysis of indices of respiratory activity and EDA electrophysiological measures provide further information on the state of autonomic regulation in terms of breathing patterns and sweat-sensitive skin conductance [45]. Trained specialist staff will perform the measurements, lasting up to 30 min, in a quiet room on the clinic campus. After a brief greeting and explanation of the procedure, the measurement equipment is applied to the participant, who is asked to sit quietly and not to speak during the recording. Speech and movement could otherwise impair the quality of the recorded electrophysiological measures or even make them unusable. The aim is to capture at least 15 min of good quality continuous recording. At the end of the appointment, the electrodes and measuring devices are removed and the participant is discharged. The following electrophysiological measures are derived in resting seated position utilizing a Somnomedics SOMNO HD device:

- Common electrophysiological measures monitoring device: Electrocardiography (3-channel ECG), respiration activity (respiration-belt), pulse wave (finger clip PPG / photoplethysmography), and electrodermal activity (EDA, finger clips).
- Additional Corsano wearable (bracelet): pulse rate (heart rate), pulse wave (PPG), derivative respiration activity, and EDA.

Based on this methodology, the individual physical (stress) state is to be measured and the *LAGOM* intervention program is evaluated using objective electrophysiological parameters in addition to the self-reports [46–49]. It is hypothesized that the intervention leads to more resilience, better mental balance, and improved stress management. We therefore expect improvements in (subliminal) stress markers that are reflected in the state of autonomic regulation of *LAGOM* participants. The measurements are accompanied by simplified derivations using wearables, which are primarily intended to test the handling and signal quality of these devices. With sufficient data quality compared to conventional monitoring devices, such wearables could offer an easier way for electrophysiological measures and ANS evaluations in the future. Due to the complexity of the electrophysiological measures analyses, the underlying approaches and methods will be described and published in more detail in a separate results paper once the study has been completed.

### Recruitment

**Fig. 2:**
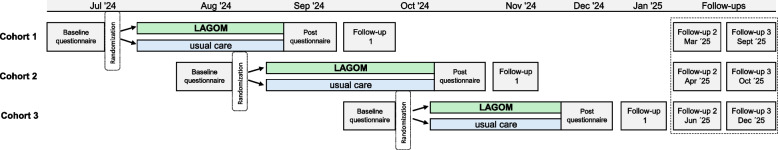
Planned timeline

Recruitment is carried out in various ways. The study steering committee, which also consists of the commercial center management and clinic directors, advertises the study to its own employees and encourage participation. The *LAGOM* program is also presented at various management events. The content of the program is presented to managers and parts of the program are offered for self-experience on site. The *LAGOM* website, which is anchored on the Charité – Universitätsmedizin Berlin sustainability homepage (https://nachhaltigkeit.charite.de/gesundheit/lagom/), also provides information about the study and promotes the program. *LAGOM* employees go to the various wards to promote the study as required. All potential study participants are informed that participation is working time. The information sessions and the website give prospective participants detailed information about the study and consent procedures. Eligible participants are informed about the study procedure and receive written study information, which they can take home and review at their leisure. A few days later, they are contacted by the study nurse, who is responsible for the inclusion of participants, by telephone and asked whether they would like to take part in the study. During this phone call, any unanswered questions are clarified. Interested participants must then submit a written informed consent to the study nurse and complete the baseline questionnaire in order to be included in the study. The reasons for non-participation are recorded. As shift planning is usually finished at least 2 months in advance, recruitment of participants will start in the month before for the cohorts respectively. All participants are informed that they can withdraw from the study at any time without giving reasons and that this will have no negative consequences for their job. Due to the limited group sizes, participants take part in the *LAGOM* program in three cohorts: One starting in July, one in August and one in October 2024. Figure 2 shows the planned participant timeline.

### Inducements for participation

LAGOM takes place during working hours. There are no further inducements for completing the *LAGOM* program. Further, as this is a pragmatic study, there is no adherence optimization method in order not to interfere with or modify routine practices for intervention delivery.

### Randomization and blinding

The randomization process is a computerized randomization sequence, automated, and centralized via the online survey tool SoSci Survey—a white-label online questionnaire tool that adheres to the requirements of German data privacy laws with servers and operators based in Munich, Germany. After agreeing to participate and signing written informed consent, participants receive a link to the online questionnaire on SoSci by e-mail. After completing the baseline questionnaire, the program automatically randomizes the participants. Randomization takes place continuously due to the temporally delayed start of the cohorts. Participants are stratified by profession (nurses vs. physicians) and blocks are the two participating centers (Charité vs. Immanuel). A distribution of 2:1 (nurses:physicians) is aimed to ensure the interdisciplinarity of the groups. This may not be feasible for practical reasons, in which a different distribution of the group composition is accepted. Participants are informed of their allocation individually via personalized email together with all necessary information regarding the upcoming steps. The emails are sent by a study nurse who is not involved in the data analysis. Even though a contamination of both intervention groups is possible, clustering at the level of the centers is not feasible due to the limited numbers of participating centers. Hence, participants are randomized at the individual level. Due to the nature of the intervention, participants cannot be blinded. The data analysts is blinded to study group, outcomes are self-assessed by healthcare professionals.

### Data analysis

The online questionnaire is implemented using SoSci Survey [50] and made available to participants via https://survey.charite.de, a licensed server of the Charité –Universitätsmedizin Berlin. Data on SoSci is collected and pseudonymized, so that an assignment of baseline questionnaires to post-questionnaires but no identification of individuals is possible.

Descriptive statistics are computed for the overall population and by group. Descriptive statistics are presented as mean ± SD, 95% CI for continuous data, and as absolute numbers and relative frequencies (percentages) for categorical variables. Two-way repeated-measures analysis of variance (rmANOVA) is used to analyze data. The factors are group (*LAGOM* vs. control) and time (baseline, post-treatment week 10, and follow-up 1 week 14). The primary outcome analysis (emotional exhaustion) refers to the time × group interaction. Group and time effects are reported as well. Partial eta-squared (η^2^p) is reported as a measure of effect size. Post hoc analysis with Bonferroni correction is performed. Secondary outcomes are evaluated exploratively using rmANOVA with no adjusted *p* values for multiple testing. Two analysis sets are used: An intention to treat (ITT) set that includes all randomized participants and a per protocol (PP) set that includes all participants that received at least 50% of the intervention. If missing at random, missing values are replaced by multiple imputation methods generating and averaging 50 additional data sets. No interim analyses are planned. The primary outcome is analyzed using the ITT set. PP analyses will be reported as well. Explorative subgroup analyses are carried out in a hypothesis-generating manner using the *R subscreen* package. All other analyses are performed using the Statistical Package for Social Sciences software (IBM SPSS Statistics for Windows, release 29.0; IBM Corporation, Armonk, NY). A *p*-value < 0.05 is considered significant. The electrophysiological measures are processed using MATLAB (MATLAB version: 9.14.0 (R2023a); The MathWorks Inc.; Natick, Massachusetts).

### Rating of pragmatism: PRECIS-2 toolkit

**Fig. 3:**
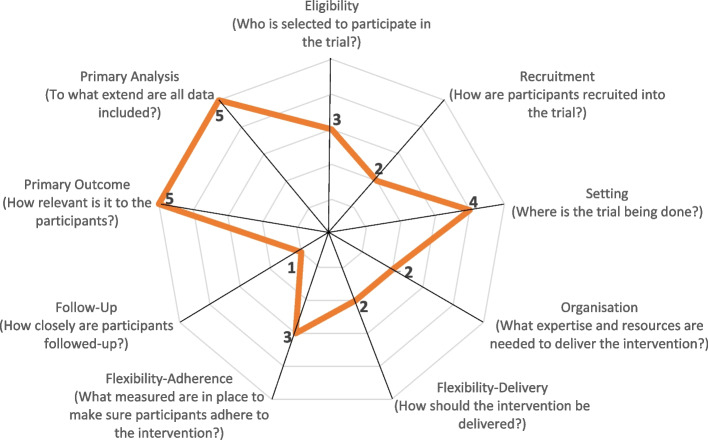
PRECISE rating

We use the Pragmatic-Explanatory Continuum Indicator Summary (PRECIS)-2 toolkit [51] to assess how pragmatic the planned study is. The tool comprises nine domains: Eligibility criteria, recruitment, setting, organization, flexibility (delivery), flexibility (adherence), follow-up, primary outcome, and primary analysis. Three members of the research team rated these nine domains on a scale of 1 to 5, with 5 being the most pragmatic rating. If the assessors gave different ratings, the ratings were discussed until all assessors agreed on a rating. The rating is based on the assessment of how easily the results of the study can be applied in a similar situation/environment. A score of 5 therefore means that the new procedure could easily be implemented in a specific real-life context. Figure 3 shows the consented PRECIS-2 rating. Primary analysis and primary outcome are rated as “very pragmatic,” setting as “rather pragmatic,” eligibility and adherence as “equally pragmatic and explanatory,” delivery, organization and recruitment as “rather explanatory” and follow-up as “very explanatory.”

### Power and sample size

The *R* package 'pwrss' is used to calculate the sample size for the interaction effect time x group using repeated measures ANOVA [52]. Emotional exhaustion is the primary outcome. Considering two repeated measurement time-points (post-intervention and follow-up 1) and two groups (*LAGOM* vs. control), an alpha error of 0.05, and a statistical power of 80%, a minimum of 70 participants is required to detect an effect size of Cohen’s *d* = 0.34. Effect size assumptions are based on statistical parameters reported within meta-analyses of 35 years of burnout prevention research [19, 20]. Hence, our study is powered to detect a small effect size for the primary outcome measure emotional exhaustion. There is evidence that 1-point changes in burnout scores are associated with significant differences in important negative outcomes such as suicidal ideation [3–6]. Even small effects in terms of emotional exhaustion should therefore result in a relevant and noticeable change. The assumptions based on the meta-analyses are also confirmed by our own preliminary study (manuscript in preparation). Considering a 20% dropout rate, the sample size required for this study is 84 participants (42 per group).

### Data management

The PCs of the departments involved are each set up with a personal password and a screen saver with password entry on reactivation. This prevents potential access to data by third parties during short absences of employees. An anonymized copy blind to which arm will be handed to the statistician (AKK) who will conduct the primary analysis. During the trial the statistician is excluded from information that would help identify the two study groups. Study participants can withdraw their consent to participate in the study at any time without giving reasons and without adverse consequences. They can also withdraw consent to the further processing of collected data and request its deletion. Only the core team of the trial will have access to the data.

## Discussion

This paper describes the protocol of the *LAGOM* effectiveness evaluation. *LAGOM* is a customized, evidence-based, theory-driven burnout prevention intervention for healthcare professionals. The program is developed together with the healthcare professionals and various advisory boards using the IMA [25]. The aim is to create a sustainable, health-promoting and meaningful working environment in hospitals so that work creates happiness and employees experience job satisfaction and remain healthy in the long term. This is not just about the absence of stress, but about experiencing meaningfulness, purpose, vitality, appreciation, and a sense of belonging. Teamwork is a decisive factor in this context. Good teamwork is highly important because it increases efficiency, creativity, and motivation, which leads to better results in patient care [53]. To this end, *LAGOM* integrates behavioral and contextual prevention. Hospitals in Germany are legally obliged to offer effective preventative measures for mental health. However, due to complex framework conditions, this is particularly difficult for nursing staff and physicians to implement in everyday clinical practice. However, it is precisely these occupational groups that urgently need support in coping with the high demands that the profession places on them. When developing prevention programs, it is therefore essential for success to actively involve the intervention participants in the development of the intervention from the outset. Without the involvement and active support of various people at management level, it is almost impossible to implement such prevention programs during working hours. For *LAGOM*, the management of the two hospitals, the clinic, and nursing directorates have supported the project in all phases and make it possible for employees to participate during working hours by planning their duty schedules at an early stage. During the piloting of the program (manuscript in preparation), it became clear that early consultation with the relevant ward managers is essential in order to be able to coordinate participation in LAGOM and the hospital routines.

Burnout is furthermore a multidimensional construct that is largely caused by poor working conditions. Behavioral prevention alone is therefore not very effective [20–23]. Structural prevention should always be part of the preventive measures, with the aim of achieving a sustainable structural improvement in working conditions for the healthcare professions. *LAGOM* takes this into account.

The results of the RCT will be published in peer-reviewed journals applying international authorship eligibility guidelines as well as at international conferences for a broad public. Professional writers are not involved. In addition, the various stakeholders at Charité Universitätsmedizin Berlin and Immanuel Hospital will be informed about the progress and results of the study at various points in time, with the aim of integrating the *LAGOM* program into the prevention services of these and other hospitals in the long term.

## Trial status

Protocol version Nr. 1, 31st May 2024. Participant recruitment began on 03th June 2024. Planned completed recruitment is 01st October 2024.

## Supplementary Information

Supplementary Material 1.

Supplementary Material 2.

## Data Availability

Not applicable.

## Abbreviations

ANS: Autonomic nervous system
HRV: Heart rate variability
IMA: Intervention Mapping Approach
ITT: Intention to treat
LAGOM: Longterm Approach and Guidelines for Occupational Mental Health with MBM
MBM: Mind-body medicine
PP: Per protocol
PRECIS: Pragmatic-Explanatory Continuum Indicator Summary
PWV: Pulse wave variability
RCT: Randomized controlled trial
