# Supplementary material for: A custom tailored, evidence-based, theory-informed intervention for healthcare professionals to prevent burnout (LAGOM): study protocol for a pragmatic randomized controlled trial
Source: Trials. 2024 Sep 27;25:628. doi: 10.1186/s13063-024-08491-1 (PMC11429380; doi:10.1186/s13063-024-08491-1)
Supplement: Supplementary file 2 — Supplementary Material 2. [file 13063_2024_8491_MOESM2_ESM.pdf]

Charité | Augustenburger Platz 1 | 13353 Berlin

Klinik für Pädiatrie m. S. Onkologie & Hämatologie

Direktorin
Prof. Dr. med. A. Eggert, MBA

AG Prävention, Integrative Medizin und Gesundheitsförderung in der Pädiatrie

Arbeitsgruppenleiter:
Prof. Dr. med. Georg Seifert

Postadresse
Campus Virchow-Klinikum
Augustenburger Platz 1 | 13353 Berlin

Einwilligungserklärung
Version 2, Stand 13.05.2024

LAGOM-Wirksamkeitsstudie (LAGOM: LAngfristige Gesundheitsbezogene Organisationskonzepte mit Mind-Body-Medizin)

Burnout-Präventionsprogramm für Mitarbeiter*innen der Pflege und Ärzteschaft

Hiermit erkläre ich (Vorname, Name) ___________________________________, dass ich mündlich und schriftlich über das Wesen, die Bedeutung und die Risiken der wissenschaftlichen Untersuchungen im Rahmen der o.g. Studie informiert wurde und ausreichend Gelegenheit hatte, meine Fragen mit dem*r aufklärenden Studienmitarbeiter*in zu klären.

Mir ist bekannt, dass ich das Recht habe, meine Einwilligung jederzeit ohne Angabe von Gründen und ohne nachteilige Folgen für mich zurückzuziehen sowie einer Weiterverarbeitung meiner Daten widersprechen und ihre Vernichtung verlangen kann. Ich habe eine Kopie der schriftlichen Studieninformation und der Einwilligungserklärung mit Versions-Datum 13.05.2024 erhalten.

Ich erkläre, dass ich freiwillig bereit bin, an der wissenschaftlichen Studie teilzunehmen.

Einwilligung in die Verarbeitung meiner personenbezogenen Daten:

Mir ist bekannt, dass bei dieser Studie personenbezogene, insbesondere auch sensible Daten zu meiner Gesundheit, zum kulturellen Hintergrund o. ä. auf Grundlage meiner freiwilligen Einwilligung verarbeitet werden sollen.

Ich wurde anhand des Informationsblattes ausführlich und verständlich darüber aufgeklärt, dass meine in der Studie erhobenen Daten, insbesondere Angaben über meine Gesundheit und soziodemographische Variablen zu den in dem Informationsblatt zur Studie beschriebenen Zwecken erhoben und in pseudonymisierter Form gespeichert und ausgewertet werden. Ich bin damit einverstanden, dass die Studienergebnisse in anonymer Form veröffentlicht

werden dürfen und dass bei medizinischen Journalen die Datensätze ohne Pseudonym oder mit einem neuen Pseudonym hinterlegt werden dürfen.

Mir ist bekannt, dass ich von der Studienleitung jederzeit Auskunft, die Berichtigung und die Löschung meiner Daten verlangen kann. Hierzu wende ich mich an die Studienleitung, die meine Daten re-identifizieren kann. Außerdem kann ich Beschwerde bei einer Datenschutzbehörde einlegen.

Insbesondere auch einer Anonymisierung meiner personenbezogenen Daten zum Zwecke der Veröffentlichung stimme ich zu. Mir ist bewusst, dass das Anonymisieren dazu führen kann, dass eine Rückverfolgung der Datenverarbeitung ausgeschlossen ist, sodass ich meine Rechte auf Auskunft, Berichtigung oder Löschung nicht mehr durchsetzen kann.

Ich wurde darüber aufgeklärt, dass ich meine Einwilligung in die Datenverarbeitung jederzeit für die Zukunft widerrufen kann und dass der Widerruf die Rechtmäßigkeit der bereits erfolgten Datenverarbeitung nicht berührt. Der Widerruf hat zudem keine Auswirkungen, sollte hierdurch die Durchführung des Forschungsvorhabens unmöglich oder ernsthaft beeinträchtigt werden.

Ich stimme der Erhebung und Verarbeitung meiner personenbezogenen Daten durch die Studienleitung zum Zwecke der Durchführung der Studie somit freiwillig zu.

____
Datum, Unterschrift

Eine Ausfertigung der Informationen und der Einwilligungserklärung habe ich erhalten.
Im Rahmen eines Aufklärungsgesprächs hatte ich die Gelegenheit hierzu Fragen zu stellen. Für meine Entscheidung hatte ich ausreichend Zeit.

Hiermit erkläre ich, den Teilnehmer bzw. die Teilnehmerin am .... über Wesen, Bedeutung und Risiken der o.g. Studie mündlich und schriftlich aufgeklärt, alle Fragen beantwortet und ihm bzw. ihr eine Kopie der Studieninformation und der Einwilligungserklärung übergeben habe.

Berlin, den

....
Name — Unterschrift der aufklärenden Studienmitarbeiterin bzw. des aufklärenden Studienmitarbeiters
